# Calprotectin (S100A8/S100A9) and Myeloperoxidase: Co-Regulators of Formation of Reactive Oxygen Species

**DOI:** 10.3390/toxins2010095

**Published:** 2010-01-20

**Authors:** Arne Bøyum, Knut Kristian Skrede, Oddvar Myhre, Vivi-Ann Tennfjord, Christine Gran Neurauter, Helge Tolleshaug, Eirunn Knudsen, Per Kristian Opstad, Magnar Bjørås, Haakon B. Benestad

**Affiliations:** 1 Norwegian Defence Research Establishment, Division for Protection, P.O.B. 25, 2027 Kjeller, Norway; Email: Oddvar.Myhre@ffi.no (O.M.); tenv@sb-hf.no (V.-N.T.); Per-Kristian.Opstad@ffi.no (P.K.O.); 2 Institute of Medical Microbiology, National Hospital, 0027 Oslo, Norway; Email: Christine.Gran@rr-research.no (C.G.N.); Magnar.bjoras@rr-research.no (M.B.); 3 Department of Molecular Biosciences, University of Oslo, P.O. Box 1041 Blindern, NO-0316 Oslo, Norway; Email: Helge.Tolleshaug@online.no (H.T.); 4 Department of Physiology, Institute of Basic Medical Sciences, University of Oslo, P.O.B. 1103 Blindern, N-0317, Oslo University of Oslo, Norway; Email: eirunn.knudsen@medisin.uio.no (E.K.); h.b.benestad@medisin.uio.no (H.B.B.)

**Keywords:** chemiluminescence, polymorphonuclear neutrophils (PMN), myeloperoxidase, calprotectin, S100A8/A9, NaOCl, albumin, cytidine deaminase, 4-hydroxy-benzoic acid

## Abstract

Inflammatory mediators trigger polymorphonuclear neutrophils (PMN) to produce reactive oxygen species (ROS: O_2_^-^, H_2_O_2_, ∙OH). Mediated by myeloperoxidase in PMN, HOCl is formed, detectable in a chemiluminescence (CL) assay. We have shown that the abundant cytosolic PMN protein calprotectin (S100A8/A9) similarly elicits CL in response to H_2_O_2_ in a cell-free system. Myeloperoxidase and calprotectin worked synergistically. Calprotectin-induced CL increased, whereas myeloperoxidase-triggered CL decreased with pH > 7.5. Myeloperoxidase needed NaCl for CL, calprotectin did not. 4-hydroxybenzoic acid, binding ∙OH, almost abrogated calprotectin CL, but moderately increased myeloperoxidase activity. The combination of native calprotectin, or recombinant S100A8/A9 proteins, with NaOCl markedly enhanced CL. NaOCl may be the synergistic link between myeloperoxidase and calprotectin. Surprisingly- and unexplained- at higher concentration of S100A9 the stimulation vanished, suggesting a switch from pro-oxidant to anti-oxidant function. We propose that the ∙OH is predominant in ROS production by calprotectin, a function not described before.

## 1. Introduction

During phagocytosis of microorganisms polymorphonuclear neutrophils (PMN) rapidly and heavily increase oxygen consumption, mainly due to production of reactive oxygen species (ROS) [1]. This respiratory burst, which can be triggered from various surface receptors of PMN, can be measured as chemiluminescence (CL) in a luminometer. During ROS formation oxygen is reduced to superoxide (O_2_^-^), which is converted to H_2_O_2_, either spontaneously or by the catalytic action of superoxide dismutase. Further reactions generate two potent reaction products, hydroxyl radicals (∙OH), in the presence of iron, and hypochlorous acid (HOCl), catalysed by myeloperoxidase (MPO) and chloride ions. In neutrophils approximately 30-40% of the detectable superoxide is converted to HOCl [2]. Unfortunately, ROS not only kill microbes, but they can also cause tissue damage by modifying DNA, lipids, proteins, and carbohydrates [1,3]. It is therefore necessary to control the cellular redox state to balance beneficial and harmful effects of these agents.

MPO, the catalyst of HOCl production, may amount to 5% [4] of total protein in PMN, being mostly present in the primary granules [5]. We wanted to examine whether the most abundant cytosolic PMN protein, calprotectin, might interact with this important generator of ROS. Another cytosolic protein, cytidine deaminase, was chosen as control.

Calprotectin [6,7] is a heterodimer composed of 11 and 13 kDa subunits, whose precise functions are still unknown. It belongs to the S100 [8] (S100A8/S100A9) protein family [9]. This calcium-binding protein with antimicrobial activity constitutes about 5% of total PMN protein [7] and as much as 40-50% of cytosolic protein [10]. Calprotectin takes part in inflammatory processes [11], but it is not known how. Increased concentrations of calprotectin, extracellularly in various body fluids and probably released by PMN, are found in many types of infectious diseases [7].

Cytidine deaminase has, apart from its enzyme function, a suppressive effect on hematopoietic colony formation *in vitro* [12,13]. One granulocyte contains approximately 0.1 pg cytidine deaminase [14], which may correspond to 0.3-1.0% of cellular protein extractable with water. Plasma concentrations of cytidine deaminase increase markedly in response to physical stress [14] and during sepsis [15].

Since calprotectin and cytidine deaminase co-exist with MPO in PMN, it is difficult to evaluate their individual contribution to ROS production in intact cells-if any. Therefore, we used the MPO-H_2_O_2_-system as a cell-free model of the respiratory burst [16,17], to examine the effects of added calprotectin or cytidine deaminase. Luminol was used as effective amplifier of MPO-dependent CL. We compared ROS-induction by MPO and calprotectin acting alone, but also together and by varying pH, temperature, and several putatively enhancing or inhibiting ingredients. The results were compared with responses elicited by intact PMN and macrophages.

## 2. Results

### 2.1. Basic response pattern

**Figure 1:**
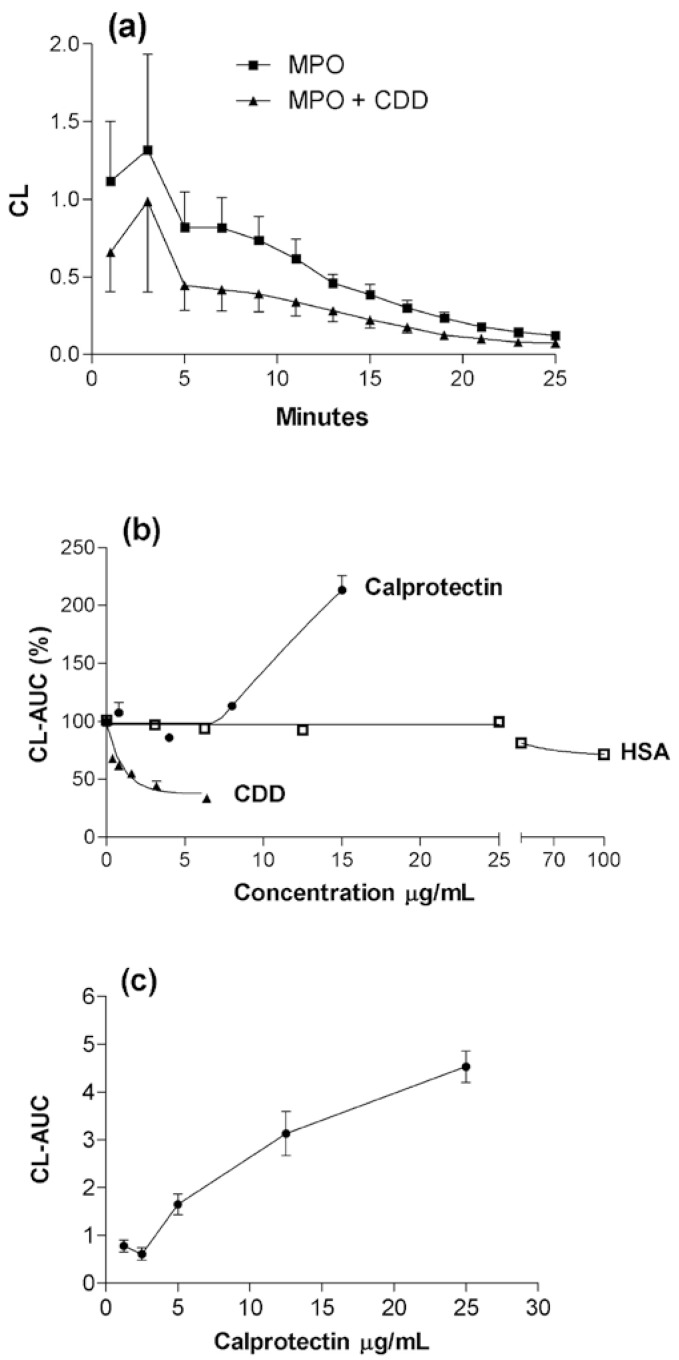
Luminol-dependent chemiluminescence (CL) of myeloperoxidase (MPO) is modified by addition of calprotectin, cytidine deaminase (CDD), and human serum albumin (HSA). CL was measured in duplicate every minute (not all values plotted) for 25 minutes at pH 7.4. (a): Time-response pattern obtained with a mixture of H_2_O_2_ (500 µM) + MPO (0.1 µg/mL) in HBBS (120-130 mM NaCl), ±CDD (3.2 µg/mL). The CL was measured as relative light units. Mean values with SEM (sometimes hidden in the symbols) from five experiments. (b): CL was induced as described in (a), and the effects of calprotectin, CDD and HSA are presented as area under the 0-25 minutes curve (CL-AUC). The values are given in per cent of MPO alone control. The combined effect of MPO and calprotectin was measured in duplicate in four experiments, of cytidine deaminase and HSA in triplicate in three parallel experiments. The percent inhibition by CDD gave almost identical results (three experiments) when carried out at room temperature and 37 ^o^C. (c): CL-AUC, dose-response curve for calprotectin (without MPO); H_2_O_2_ 500 µM, pH 7.8. Two experiments.

The cell-free H_2_O_2_-MPO-Cl^‾^-luminol system was designed to model the respiratory burst. The CL peaked within a few minutes, then tapered off for the next 23 minutes of recording (Figure 1a). Calprotectin increased (p < 0.05) CL (Figure 1b), whereas cytidine deaminase inhibited it already after one minute (p < 0.05). 

In the four experiments depicted in Figure 1 MPO (0.1-0.3 µg/mL) and calprotectin (16 µg/mL) in combination had an additive effect on light emission (p < 0.05). A moderate increase (21%) was also observed for 8.0 µg/mL calprotectin (p < 0.05), provided that the data for only the first 10 minutes were used. For a total of nine experiments the effect was not only additive (p < 0.001), but also potentiated synergistic (p < 0.01); the average CL integrals being MPO 8.9, calprotectin 4.8 and MPO + calprotectin 19.1. Even though stimulation by calprotectin varied considerably (see below), it was not significantly affected by addition of, or preincubation with, calcium (1-2 mM). It is noteworthy that PMN may contain >10.000 µg/mL calprotectin [7].

Dose-response experiments showed that the CL was enhanced more than linearly with increased concentrations of MPO (0.1-0.4 μg/mL), and at high activity of MPO (not shown) the inhibitory effect of a constant cytidine deaminase concentration (3.2 µg/mL) was reduced (p < 0.01). On the other hand, with a constant MPO concentration, cytidine deaminase exerted increasing inhibition in the dose range 0.8-6.4 µg/mL (Figure 1b). The PMN concentration of cytidine deaminase has been measured to be 100-200 µg/mL [14].

In the same molar or mass concentration ranges used for cytidine deaminase and calprotectin, control human serum albumin (HSA) had no effect on MPO-CL, but inhibited the response at concentrations ≥100 µg/mL (p < 0.05). CL was not induced with MPO or calprotectin when luminol was replaced by lucigenin (0.1 mM), which is supposed to mostly reflect superoxide formation. The calprotectin had no detectable peroxidase activity in the ECL assay.

**Figure 2:**
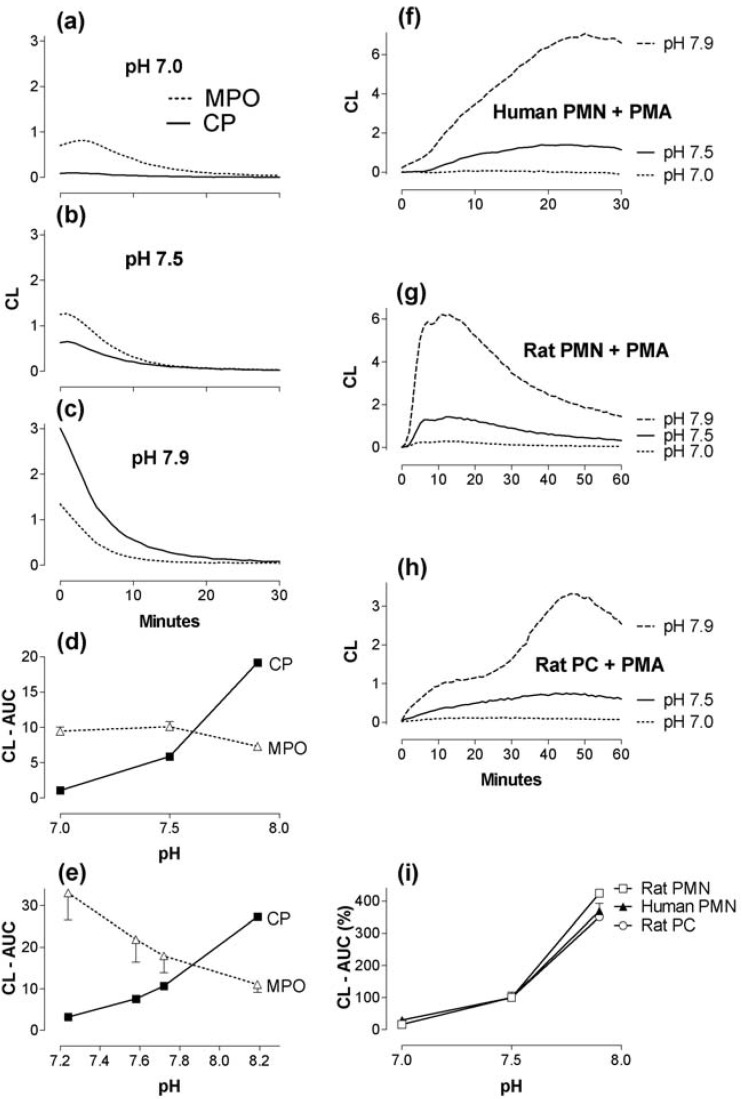
The effect of pH (a-e) on CL elicited by H_2_O_2_-stimulated (500 µM) MPO (0.1 µg/mL) and calprotectin (CP; 8 µg/mL); comparison with PMA-evoked cell responses (in phosphate buffers, 80-85 mM, osmolality 290 mOsm/kg). (a-c): Triplicate measurements of MPO- and CP-induced CL in two experiments with pH 7.0, 7.5 and 7.9 show the kinetics of the response during 30 minutes and (d) gives the corresponding AUC (e): CL elicited by MPO and CP in Dulbecco’s phosphate-buffered (~6 mM) saline (~105 mM NaCl)-pH adjusted with NaOH (f): CL elicited by PMA-stimulated human granulocytes (2 × 10^5^/well). (g): rat granulocytes (1.5 × 10^5^/well) and (h): rat peritoneal cells (PC; 3 × 10^5^/ well). PMA was 10^-7^ M for human cells and 4 × 10^-7^ M for rat cells. Mean values from two experiments. The graphs (f, g, h) show CL responses for three different pH values and the integrals (AUC) are shown in (i), in per cent of integrals obtained with pH 7.5. The pH (except e) was adjusted by appropriate portions of Na_2_HPO_4_ and NaH_2_PO_4_ (85 mM phosphate, 63 mM NaCl).

### 2.2. The effect of pH changes on MPO- and calprotectin-chemiluminescence, and responses by granulocytes and peritoneal cells

As demonstrated for the first time here, calprotectin itself can induce CL. However, the activity of calprotectin was lower than for MPO, and we wanted to test if it could be enhanced by modifying the experimental procedure. At first we tested pH changes and found that the response (AUC) to calprotectin (8 µg/mL) was increased from 1 to 19 (p < 0.001) and the MPO-dependent response decreased (AUC from 10 to 7.3, p < 0.01) along with elevation of pH from 7.0 to 7.9 (Figure 2a-d). In four new experiments these results were confirmed with another buffer (Dulbecco’s phosphate-buffered saline), and calprotectin luminescence could be markedly enhanced by a pH increase from 7.1 to 7.4. In additional tests we found that 2-4 µg/mL of calprotectin, with pH 7.9 and 60-500 µM H_2_O_2_ was appropriate for routine use. 

Controls, relevant according to molar and mass concentrations, with HSA (up to 1000 µg/mL) or human immunoglobulin (20 µg/mL) yielded CL (AUC) of ~1 or less (not shown). A pH-dependent pattern was also observed for CL elicited by PMA-stimulated human granulocytes, rat granulocytes, and rat peritoneal cells (Figure 2f-h). This response (Figure 2i) resembled the pH dependency of the calprotectin-response more than that of the MPO-response (Figure 2d, Figure 2e).

**Figure 3:**
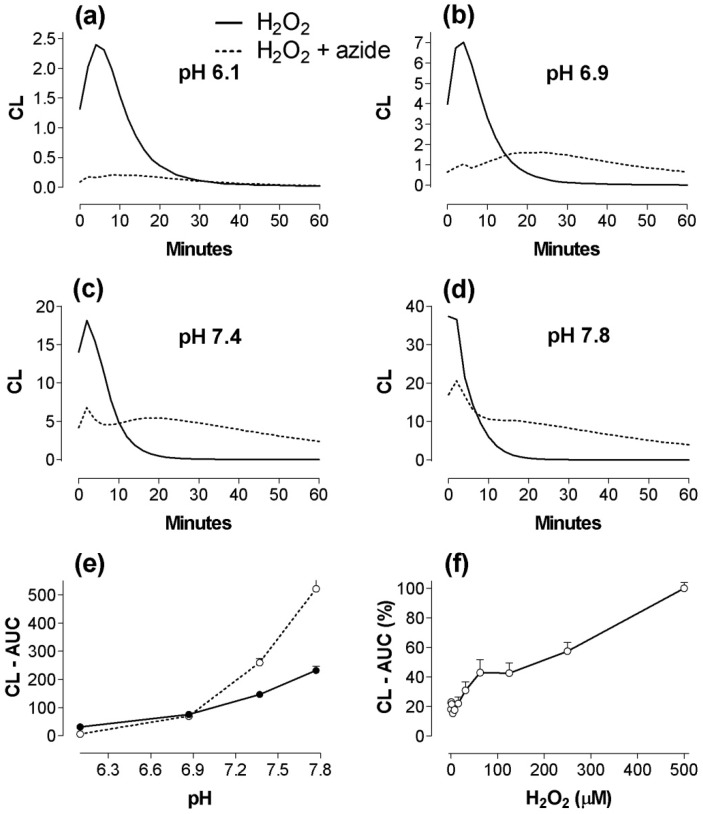
The effect of pH on kinetics of H_2_O_2_-mediated (500 µM) CL-responses in human PMN (2 × 10^5^/well) monitored for 60 minutes in phosphate buffer, without and with 1 mM Na-azide (a-d). Triplicate measurements from three experiments. (e) gives the 60-minute integrals (AUC) as a function of pH. (f): Dose-response curve for H_2_O_2_ when human PMN (2 × 10 ^4^/well) were examined for 30 minutes in a buffer with pH 7.8. Mean values from three experiments in per cent of CL-AUC with 500 µM. Note different scales on Y axes (a-d).

This pattern was confirmed when H_2_O_2_ instead of PMA was used as stimulator of human PMN (Figure 3), thereby presumably bypassing the contribution by phagocyte NADPH oxidase. This H_2_O_2_ response was almost aborted by azide (1 mM) at pH 6.1, and the 12-minute response was consistently reduced at pH 6.9-7.8, probably reflecting an inhibitory effect of azide on the heme enzyme (MPO). However, as pH increased, the response in the azide groups was changed (Figure 3a-d), and the 60-minute CL integral (at pH 7.4 and 7.8) was increased above control without azide (Figure 3e, p < 0.01). With 20000 PMN/well (Figure 3f), we found that 16 µM of H_2_O_2_ was sufficient to yield a significant (p < 0.05) response.

### 2.3. CL responses of calprotectin and MPO to H_2_O_2_, azide, NaCl, melatonin, and cysteine supplements to the reaction mixtures

**Figure 4:**
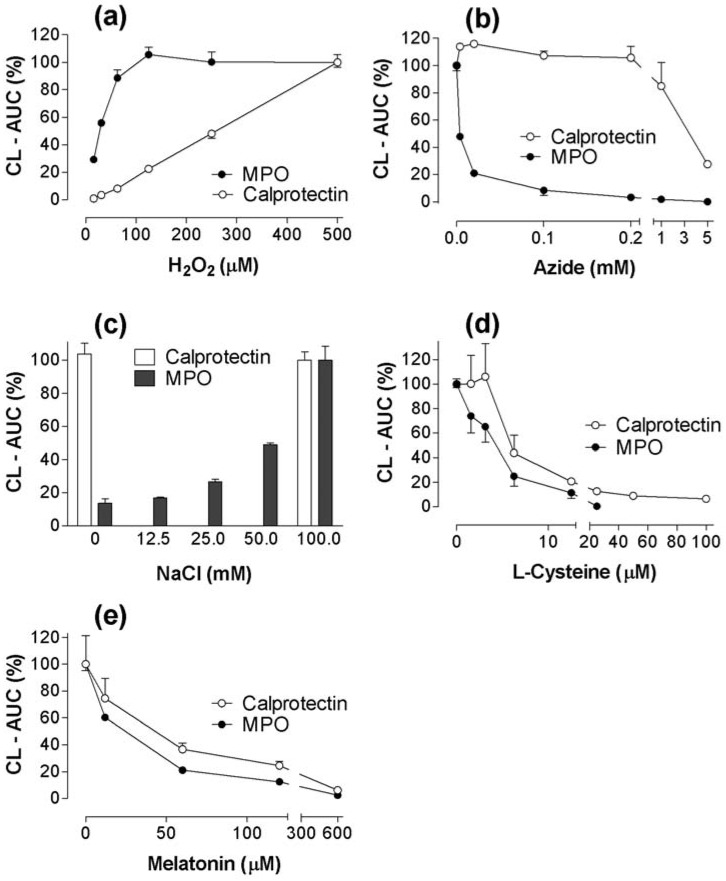
Comparative studies of MPO and calprotectin. Triplicate measurements during 30 minutes. (a): Dose-response of H_2_O_2_-induced MPO-CL and calprotectin-CL. The values are given in per cent (with SEM) of groups with 500 µM H_2_O_2_. Phosphate-buffer with pH 7.8. Mean values from two experiments. (b): Dose-response of Na-azide. MPO (0.16 µg/mL, ~1.3 × 10^-8^ M). Calprotectin (8 µg/mL, ~0.4 × 10^-6^ M). Phosphate-buffered saline, pH 7.4. The values from two experiments are given in per cent of controls (±SEM) without azide. (c) The effect of NaCl on luminol-dependent MPO-CL and calprotectin-CL. MPO (0.1 µg/mL, pH 7.4). Calprotectin (4 µg/mL, pH 7.8). The calprotectin had been dialysed against a NaCl-free solution. The values are given in per cent of groups with 100 mM NaCl. Five experiments with and without 100 mM NaCl. (d): Dose-response of cysteine on MPO-CL and calprotectin-CL (2 experiments). (e): Dose-response of melatonin on MPO-CL and calprotectin-CL (2 experiments). MPO (0.05 µg/mL, pH 7.4), calprotectin (4 µg/mL, pH 7.8) in (d) and (e).

We also wanted to compare responses of MPO and calprotectin to different reactants, some of them known to affect MPO. The responses of MPO and calprotectin to increasing concentrations of H_2_O_2_ were very different (Figure 4a), even though both agents showed H_2_O_2_-dependency. H_2_O_2_ (500 µM) + luminol, alone, yielded a slight response (AUC~0.9) only at high pH (7.8). MPO was much more sensitive than calprotectin to the inhibitory effect of azide (Figure 4b). In fact, there was a slight stimulation of calprotectin-H_2_O_2_-CL at 0.02-0.1 mM azide (p < 0.05). Calprotectin-H_2_O_2_-CL was independent of the presence of NaCl, whereas MPO-H_2_O_2_-CL required a high NaCl concentration (Figure 4c), presumably for HOCl production. The response pattern was similar for inhibition of MPO and calprotectin by added cysteine and melatonin - added as anti-oxidants [18]-with MPO the more sensitive (p < 0.01) (Figure 4d, Figure 4e).

### 2.4. The combined effect of calprotectin and NaOCl

**Figure 5:**
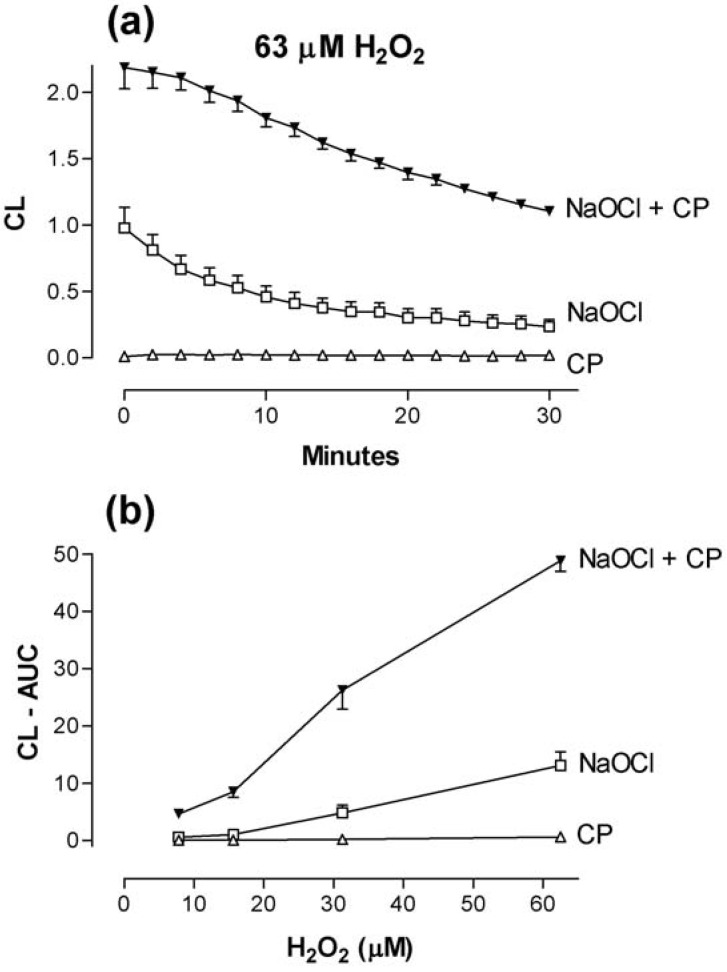
Luminol-enhanced combined effect of calprotectin (CP; 4 µg/mL) and NaOCl (20 µM) in phosphate buffer with pH 7.8. CP and NaOCl were incubated at room temperature for 17 minutes [19] before luminol and H_2_O_2_ were added to initiate ROS formation. (a): Time-course of CL responses recorded during 30 minutes with 63 µM H_2_O_2_ (b): Effect of increasing concentrations of H_2_O_2_ on CL induced by NaOCl, CP, and NaOCl + CP. Two experiments.

The combination of calprotectin (4 µg/mL) and NaOCl (20 µM) strongly enhanced CL in the presence of H_2_O_2_ (Figure 5), possibly caused by oxidation of calprotectin by NaOCl [19]. Since HOCl is the end product in the MPO-system, this finding may explain the synergy observed by combining MPO and calprotectin (Figure 1). NaOCl itself may generate significant CL, but high background values are largely avoided at low concentrations of H_2_O_2_ (Figure 5). Noteworthy, it was necessary to incubate the mixture of calprotectin and NaOCl for 17-20 minutes at room temperature in these experiments, before the CL reaction could be initiated by adding luminol and H_2_O_2_ and enhancement demonstrated [19]. Dose-response experiments showed that a calprotectin concentration of 0.5-1.0 µg/mL was sufficient, when combined with NaOCl and H_2_O_2_ (>16 µM), to induce a significant CL response, whereas no response was observed in the absence of NaOCl. 

### 2.5. Recombinant S100A8 (A8) and S100A9 (A9)

**Figure 6:**
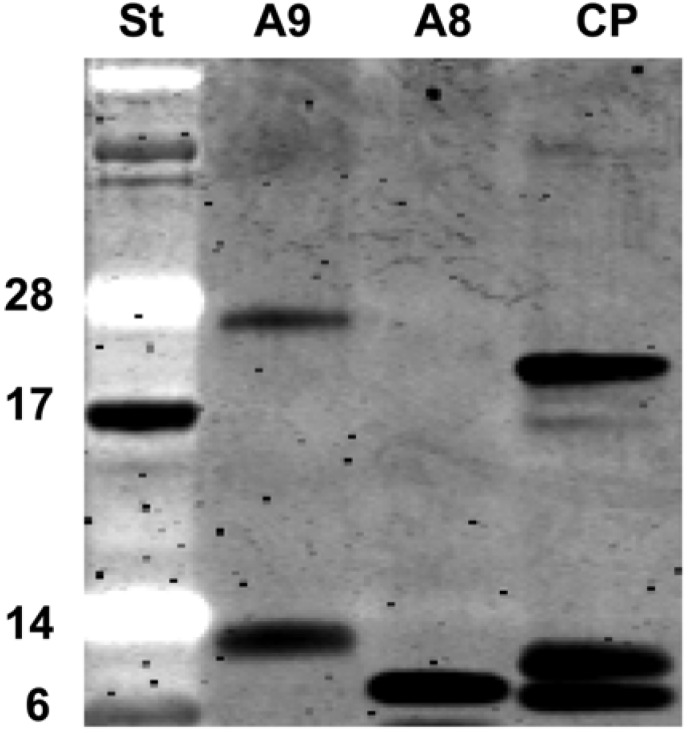
Recombinant S100A8 and S100A9, and calprotectin (CP), were separated on SD-PAGE prior to SYPRO Ruby staining. A9 tended to form homodimers, whereas with the mixture of leucocyte-derived A8 and A9 (CP), the proteins migrated as monomers, homodimers or heterodimers. Standard (St) with molecular weight markers (6, 14, 17, 28) in kDa.

A8 remained a monomer in solution while A9 formed a dimer as well (Figure 6), but to a different extent in different batches. Dimerization apparently enhanced the capacity to generate CL in the presence of H_2_O_2_ (data not shown). 

**Figure 7:**
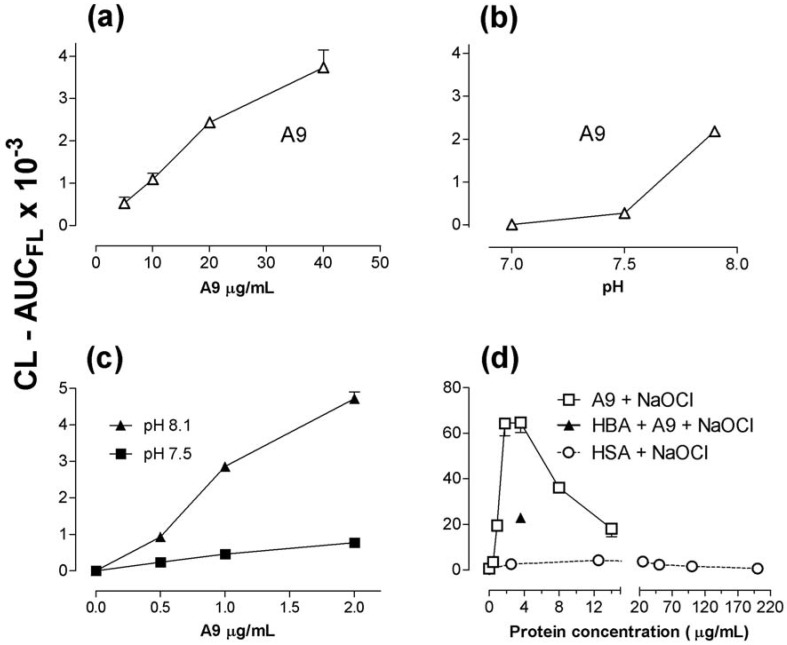
Dose-response curves and pH-dependency for CL generated by A9 and HSA in a H_2_O_2_-luminol system. (a) shows CL at different concentrations of A9 (pH 7.9); (b) shows CL induced by A9 (20 µg/mL) at three different pH. The H_2_O_2_ concentration (a, b) was 500 µM. (c) gives dose-response curves for A9 at two different pH values; 32 µM H_2_O_2_. (d) shows CL induced by A9 + NaOCl (20 µM), or HSA + NaOCl (20 µM). The effect of 4-hydroxy-benzoic acid (HBA, 2 mM ), an ∙OH trapping compound, was assayed at one A9 concentration (3.6 µg/mL). All set-ups in (d) with 63 µM H_2_O_2_. Mean values from two experiments, corrected for background values in buffers. The experiments were carried out with the FLx800 luminometer at room temperature.

The activity of A9 (Figure 7a, Figure 7b) clearly depended upon both protein concentration (5-40 µg/mL) and pH, as did A8, which produced less CL (not shown). Mercaptoethanol-treated A9 elicited no detectable CL. A9 with NaOCl + 32 μM H_2_O_2_ gave a marked pH-dependent CL-response (Figure 7c). An even stronger response was observed with 63 μM H_2_O_2_ (Figure 7d). However, increasing concentrations above 3.6 µg/mL led to a gradual decrease of the CL, in contrast to the set-up without NaOCl included (Figure 7a). Similarly, HSA + NaOCl evoked a slight increase of CL up to 25 µg/mL albumin, declining to no response at 200-1,000 µg/mL. HSA without NaOCl did not cause any CL (2.5 to 1,000 µg/mL). At pH 7.8 similar responses were observed for A8 and A9 when combined with NaOCl (not shown). The activity of mercaptoethanol-exposed A9 was also restored by NaOCl. 

**Figure 8:**
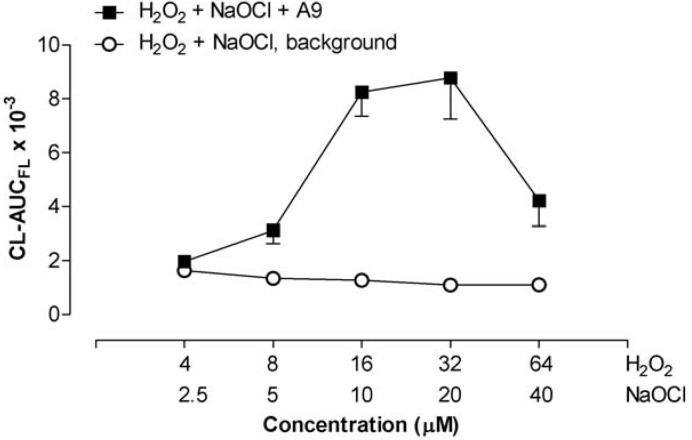
Chemiluminescence at constant H_2_O_2_/NaOCl ratio (=1.6). A9 1.2 µg/mL. Phosphate buffer with pH 7.8. Mean values with SEM from three experiments, with the FLx800 luminometer at room temperature.

A molar ratio of 1.6-3.2 between H_2_O_2_ and NaOCl proved to be optimal for A9-induced CL. We assessed the procedure by keeping the ratio constant at 1.6 in the dose ranges of 4-64 µM for H_2_O_2_ and 2.5-40 µM for NaOCl and obtained a bell-shaped curve for A9-CL, with 1.2 µg A9 per mL (Figure 8). Low background levels of the NaOCl + H_2_O_2_ were maintained. We saw A9-CL significantly above background (p < 0.05) with only 5.0 µM NaOCl + 8 µM H_2_O_2._ These values of NaOCl and H_2_O_2_ are in the physiological range [4,20,21]. It is possible to trigger ROS formation even more by increasing the H_2_O_2_ concentration (with constant NaOCl), but this tends to elevate the NaOCl-induced background levels and leads to more variable responses. For routine use, 10 or 20 µM NaOCl and H_2_O_2_ in the 16-64 µM range is recommended.

### 2.6. 4-hydroxy-benzoic acid (4-HBA) and L-histidine

4-HBA (2 mM), considered to be an agent trapping hydroxyl radicals [22,23,24], stimulated (p < 0.05) the luminol-H_2_O_2_- dependent (500 µM H_2_O_2_) MPO-response by 28% (five experiments). On the contrary, 4-HBA abrogated CL (p < 0.05) by A8 (500 µM H_2_O_2_, three experiments) and A9 (500 µM H_2_O_2_, two experiments). In five experiments 4-HBA did not affect CL elicited by NaOCl alone, but suppressed (see Figure 7d) the intense CL generated by NaOCl + A9 (1.2 µg/mL) + H_2_O_2_ (63 µM) by 65% (p < 0.001). 

**Figure 9:**
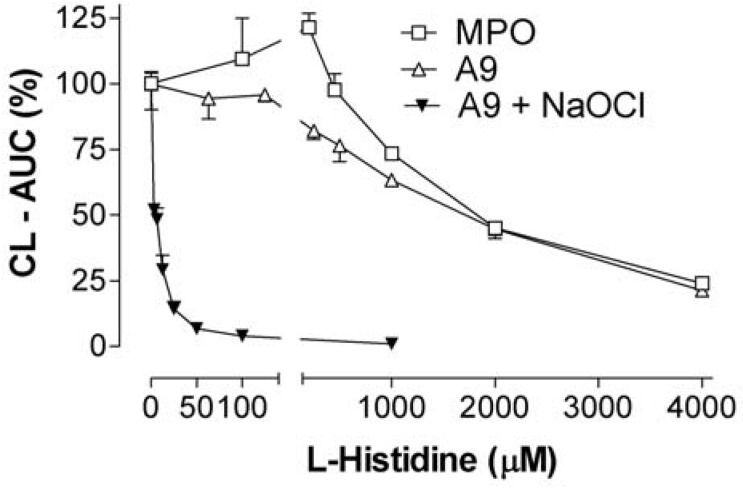
The effect of L-histidine, a putative singlet oxygen scavenger, on CL-integrals of MPO (0.05 µg/mL) and recombinant A9 (8.0 µg/mL), both with 500 µM H_2_O_2_, and A9 (0.6 µg/mL) + NaOCl (20 µM) with 63 µM H_2_O_2_. Two experiments. Values in per cent of control without histidine. The results with A9 + NaOCl were confirmed (four experiments) with the FLx800 luminometer.

Histidine, assumed to be a singlet oxygen scavenger [25], strongly inhibited the NaOCl-increased CL induced by A9, whereas inhibition was weaker in the absence of NaOCl, and then similar to the effect on H_2_O_2_-induced MPO responses (Figure 9). 

### 2.7. Temperature effects

A temperature increase from 24 to 37°C consistently enhanced CL integrals induced by A8 and A9 in combination with H_2_O_2_, but only with NaOCl in the reaction mixture (p < 0.001; data not shown). With MPO, or A9, + H_2_O_2_, we observed no consistent effect of temperature change. 

### 2.8. The effect of iron and iron chelators

In the absence of NaOCl, iron ions (FeSO_4_, 140 µM) enhanced luminol-H_2_O_2_-CL, and the effect was additive when the iron salt was combined with calprotectin. FeCl_3_ had a similar effect. Lower concentrations (10-20 µM) stimulated only marginally. When NaOCl was included, there was no additional stimulatory effect of iron ions (0.1-100 µM). Deferoxamine, an iron chelator (200 µM), significantly (two experiments) reduced the CL-response (p < 0.01) elicited by A8, A9, A9 + NaOCl, NaOCl alone, and MPO. The strongest reduction (90%) was observed with A9 + NaOCl, whereas an approximately 50% decline was obtained for MPO and NaOCl set-ups. The interpretation of these results is difficult since deferoxamine not only chelates iron, but may also bind and inactivate OH-radicals and possibly other ROS [26]. Taken together, the possibility exists that trace amounts of iron or other metals can activate calprotectin.

## 3. Discussion

We have shown that two major PMN cytosolic proteins, calprotectin and cytidine deaminase (CDD), had opposite effects on the *in vitro* luminol-amplified chemiluminescence (CL) elicited by the H_2_O_2_-MPO-Cl^-^ cell-free system. Calprotectin stimulated CL, whereas cytidine deaminase inhibited it. Calprotectin alone, without myeloperoxidase (MPO), also triggered CL. However, at pH 7.4 this effect was detectable only at concentrations of 10-20 µg/mL (~1 µM) or more, but 0.05-0.3 µg/mL (0.5-3 × 10^-9^ M) was sufficient for MPO.

We interpret the results as follows: The ROS formation by MPO and calprotectin both depended upon H_2_O_2_. Both reactions were inhibited by L-cysteine and melatonin (Figure 4). For MPO it might be due to interaction with HOCl, which is the CL promoting end product of the MPO reaction chain [2]. For calprotectin the inhibiting mechanism may be different. Calprotectin presumably does not generate HOCl, since it, unlike MPO, did not need the presence of chloride for CL (Figure 4c). Other findings indicated different CL mechanisms as well: ROS generation by calprotectin or by phagocytes (granulocytes, peritoneal cells) markedly increased with increasing pH (Figure 2), but tended to decrease in the MPO system. Comparing calprotectin and MPO we found that these two proteins also reacted differently to 4-hydroxybenzoic acid, allegedly capable of quenching hydroxyl radicals. Calprotectin had no detectable peroxidase activity as measured with the ECL assay. Taken together, these findings suggest that MPO and calprotectin stimulate CL by different mechanisms. 

The synergistic effect of the MPO-calprotectin combination (Figure 1b) may be due to MPO-induced HOCl, which stimulates calprotectin activity [19]. Notably, CL in H_2_O_2_-stimulated human granulocytes (Figure 3) looked like a two-component response, putatively exerted by MPO and calprotectin in combination. The MPO response, which seemed to predominate at low pH (Figure 3a), was almost aborted by azide, in line with the known azide-sensitivity of MPO. At increasing pH, a larger fraction of the total CL can tentatively be ascribed to the more azide-resistant calprotectin (Figure 4b).

The reduced MPO-chemiluminescence in response to a rise of pH contrasts some previous findings [27], but agrees with the results of Albert and Jungi [28]. The CL generated by intact PMN increased with pH (Figure 2 and Figure 3), in accordance with previous findings [29]. Following phagocytosis there is first a pH rise to ~ 7.8 in the phagosomes, followed by a gradual decrease towards 5-6 [30], indicating that a pH of 7.8 might be within the physiological range. It is noteworthy that a rise of pH from 7 to 8 may change the configuration and increase thermal stability of calprotectin [31].

ROS detection is based upon exitation/oxidation of luminol, which relaxes to ground state by emitting light. For granulocytes, luminol amplifies a weak luminescence that also can be observed in a luminol-free medium, supposedly without changing the pattern of ROS formation [32]. The speed of occurence and size of the CL pattern can be controlled by varying the concentrations of luminol, H_2_O_2_, and NaOCl [33,34]. In our study we found that H_2_O_2_ triggered luminol-dependent ROS formation by calprotectin (A8/A9) and this response was strongly enhanced by adding NaOCl as the fourth component. This seems to imply that calprotectin activation is linked to NADPH oxidase and MPO. In any case, a mechanism of this kind in phagocytes could probably enhance their ability to fight microorganisms. Presumably, hydroxyl radicals, generated by calprotectin, amplify the cells’ microbiocidal power. If both MPO and calprotectin can normally engage, independently of each other, creating ROS in phagocytes, this might explain why most patients who lack MPO are spared from serious infections in spite of their deficiency [35].

Clearly, there are several elements that need to be evaluated to identify the physiological role of calprotectin. Calprotectin is easily oxidized by NaOCl [19], which may modify its functional properties [36]. This points to the possibility that we may be dealing with an active redox system [37,38], with calprotectin conceivably playing a dual role during inflammation. One or the other of these roles may become the leading one, determined by environmental signals. In fact, a change of concentrations may transform reactants from pro-oxidants to anti-oxidants [37], as we observed by increasing the concentration of A9 (Figure 7d), which suggests that A9 may act as a scavenger of NaOCl. So far, most investigations have focused on inflammatory stimulation by S100A8/A9 proteins [39]. However, recent research has indicated that oxidation of these proteins may trigger a switch, whereby they come to display anti-inflammatory functions [40]. These may be enhanced by nitrosylation [41]. Furthermore, the function of A8 and A9 might be determined also by polymerization, i.e. whether they exist as momomers, homodimers, heterodimers or tetramers [42]. Proper refolding may also be important for the protein’s function. Such a mechanism could possibly explain why recombinant A8/A9 exposed to mercaptoethanol did not generate ROS. In any case this multitude of possible molecular forms could mean that the capacity to generate or scavenge ROS be present in only a small fraction of a heterogeneous multimeric complex.

It is true that a substantial part of our results are shown at an unphysiological pH and probably also hydrogen peroxide concentration. However, ours is a very artificial, simplified cell-free *in vitro* system, devised to explore possible oxidative and anti-oxidative phagocyte functions of calprotectin, as well as interactions with the better known ROS-generating cellular components. By varying concentrations of H^+^-ions, H_2_O_2_, NaOCl, and proteins we have been able to establish procedures in which the different variables are balanced within assumedly physiological limits. Whether our findings can be validated under conditions present in intact cells or in an inflammatory, extracellular environment has to be explored in further experiments. 

In the intact organism the putative interplay between MPO and calprotectin will be largely restricted to PMN, monocytes and possibly early B-lymphocytes, in which MPO gene expression has been demonstrated [43]. We suggest that on stimulation of PMN, as an initial step, O_2_^-^, generated by NADPH-oxidase, is converted to H_2_O_2_, which next may react with MPO. Its two reaction products, H_2_O_2_ and HOCl, are membrane permeable, so they might reach cytoplasmic calprotectin. Cellular H_2_O_2_ may also be generated in other metabolic pathways. For instance, electrons leaking from the mitochondria [44] lead to O_2_^-^ and H_2_O_2_ formation. In this way, calprotectin may become an integral part of cytoplasmic redox regulation or microbial defense mechanisms or both. Considering the very high calprotectin concentration in some cell types (PMN) and its widespread distribution, together with H_2_O_2_, it is hardly surprising that calprotectin has been clinically related to many disease conditions-Even in atherosclerosis [31,45]. 

## 4. Experimental Section

### 4.1. Chemicals

Hydrogen peroxide (30%), 5-amino-2,3-dihydro-1,4-phtalazinedione (luminol), bis-*N*-methylacridinium nitrate (lucigenin), myeloperoxidase (MPO), phorbol 12-myristate 13-acetate (PMA), mercaptoethanol, deferoxamine mesylate, 4-hydroxybenzoic acid, sodium azide, melatonin, L-cysteine, L-histidine and human serum albumin (HSA, MW ~ 67 kDa) were purchased from Sigma (St. Louis, USA), and Hepes buffer from BioWhittaker (Walkersville, MD, USA). Dextran 500 (Pharmacia, Uppsala, Sweden) was dissolved in 0.9% NaCl and used as a 3 or 6% solution. Sodium hypochlorite (NaOCl, 0.5 M in 0.1 M NaOH), purchased from BDH (Dorset, England), was diluted with water. Luminol and PMA were dissolved in dimethylsulphoxide (Sigma) and further diluted in water or a buffered salt solution. Phosphate-buffers of different pH were made by mixing appropriate portions of 0.2 M NaH_2_PO_4_ and 0.2 M Na_2_HPO_4_. Peroxidase was measured with the enhanced chemiluminescence method (ECL, Amersham Pharmacia Biotech, Oslo). Calprotectin, purified from human neutrophils [46], was generously provided by Axis-Shield (Oslo, Norway) and dialysed against Hanks’ balanced salt solution (HBSS, Invitrogen, Norway) or phosphate-buffered saline before use. The two subunits (S100A8 and S100A9) preferentially combine as a heterodimer, and tend to remain associated during purification [47]. However, they may also combine as homodimers and even trimers or tetramers [47], so exact characterization by MW is not always possible. Cytidine deaminase (MW~52 kDa) was produced as recombinant protein [13] and dissolved in HBSS.

### 4.2. Purification of recombinant S100A8 and S100A9

Competent *E. coli* BL21 CodonPlus(DE3)-RIL cells were transformed with pET28-S100A8 or pET28-S100A9 [48], grown in 2 L of LB medium with 25 μg/mL kanamycin to OD_600_ of 1 and induced with 1 mM isopropyl-β-D-thiogalactopyranoside (IPTG, Sigma) for 4 h at 30 ºC. Extract was prepared by sonication of the cell pellet in 40 mM Na_2_PO_4_, pH 8.0, 300 mM NaCl (sonication buffer) and cleared with centrifugation. The cell extract was applied on a Ni-column equilibrated with sonication buffer. The column was eluted with a gradient of 50-300 mM imidazole in sonication buffer, and purification of A8 and A9 (with and without mercaptoethanol) was monitored by protein gel electrophoresis. The *N*-terminal 6xHis were removed by Thrombin (T-4648, Sigma) cleavage, which enhanced the CL-inducing activity of the dialysed proteins. The purified A8 migrated as a monomer of apparent molecular weight 10 kDa, while A9 migrated as a monomer and a dimer of apparent molecular weight 13 and 26 kDa (Figure 6), respectively. Protein concentration was determined by the Bio-Rad protein assay (Bio-Rad), with BSA as a standard.

### 4.3. Chemiluminescence

A cell-free system was used as model of the respiratory burst in PMN: H_2_O_2_ + MPO + chloride + luminol. In some experiments MPO was replaced by calprotectin. The luminol-enhanced CL was measured as relative light units in a luminometer (Luminoskan, Termo Labsystems, Helsinki, Finland), reading the samples in 96-well plates (White Cliniplate, Thermo Fisher Scientific, Vantaa-Finland). In some experiments (Figure 7, Figure 8) we used a FLx800 luminometer (Biotek Instruments, Inc. Winooski, Vermont, USA). In relative terms the results were similar with the two luminometers, but the observed values (arbitrary light units) were quite different. Unless otherwise indicated, the recording was done at 37 ^o^C. Each well contained 25 µL H_2_O_2_ solution (final concentrations are indicated), 5-10 µL MPO, 50 µL luminol (0.1 mM), 10-20 µL test substance and buffer (140-170 µL) to yield a total volume of 250 µL. MPO (10-40 µg) was dissolved in 1 mL water and stored at 4 ^o^C. The activity was stable for some months, but upon further dilution it was often strongly reduced after a couple of days. The chemiluminescence was recorded (2-4 wells per group) at 1-2 minute intervals for 25-30 (or 60 minutes, Figure 3) minutes, and the integral of the response curve (AUC, area under the curve) was calculated. MPO concentration of 0.05-0.3 µg/mL mostly yielded AUC in the 5-30 range, but substantially increased responses were sometimes observed with modified procedures. Significant responses with MPO, calprotectin or cells were observed with 8-16 µM H_2_O_2_ as stimulator. However, consistent results were best obtained at higher concentration (30-500 µM). A control group with buffer, but without luminol and H_2_O_2_ was always included. Tris-buffers should be avoided because it triggers substantial CL when combined with NaOCl. 

### 4.4. Cell separation

Human blood from healthy colleagues was collected into vacutainers containing EDTA. The granulocytes were separated as described previously [49]. Male Fisher 344 rats (Møllegaard Breeding Centre, Ejby, Denmark) were also used. They were treated in accordance with institutional and national guidelines for animal research. Rat granulocytes were prepared as follows: The rats were anaesthetized with CO_2_ and killed by decapitation, and the trunk blood was collected into tubes containing EDTA. One part (3-6 mL) of EDTA-blood was mixed with one part of dextran 3% to cause aggregation and sedimentation of erythrocytes. Then 3 mL of Lymphoprep (Axis-Shield, Oslo, Norway) was installed underneath leucocyte-rich dextran-plasma (4-8 mL) in 15 mL tubes and centrifuged for 15 min at 600 g at room temperature. The bottom fraction, comprising >95% granulocytes, was collected and washed once. The cell button was suspended in 4 mL lysis solution (0.15 M NH_4_Cl, 1 mM KHCO_3_, 0.1 mM EDTA) and incubated at room temperature for 7 minutes to lyse remaining red cells. If necessary, the procedure was repeated. Rat peritoneal cells were obtained by washing the peritoneum of dead animals with 25 mL phosphate-buffered saline. The cells were pelleted by centrifugation and resuspended in phosphate buffer or HBSS supplied with 20 mM Hepes buffer (pH 7.4) and 5 mM glucose. 

### 4.5. Statistics

The results are given as means with their standard error (SEM). An analysis of variance (ANOVA) procedure was used to test for dose-response of cytidine deaminase and calprotectin. Student’s t-test was used to assess the difference between two groups. Two-sided P values < 0.05 were considered statistically significant.

## 5. Conclusions

Calprotectin (S100A8/A9), together with H_2_O_2_, induced luminol-dependent chemiluminescence (CL), and thus triggered formation of reactive oxygen species (ROS). Calprotectin and myeloperoxidase (MPO) might thereby collaborate on microbial killing. The CL responses elicited by calprotectin and MPO were differentially increased or decreased by varying the experimental conditions in several ways. Calprotectin CL was enhanced by addition of NaOCl to the reaction mixture. Surprisingly, however, at constant NaOCl concentration, this augmentation vanished when the calprotectin concentration was increased, suggesting a switch from pro-oxidant to anti-oxidant function. Possibly, calprotectin may launch an attack on microbes, but also protect our own tissues from excessive ROS exposure.

## Acknowledgements:

Expression plasmids pET28-S100A8 and pET28-S100A9 were a kind gift from Philippe Tessier [48]. We also wish to thank Trine Reistad for valuable help with establishing the CL procedure.

## References

[1] Hampton M.B., Kettle A.J., Winterbourn C.C. (1998). Inside the neutrophil phagosome: Oxidants, myeloperoxidase, and bacterial killing. Blood.

[2] Kettle A.J., Winterbourn C.C. (1990). Superoxide enhances hypochlorous acid production by stimulated human neutrophils. Biochim. Biophys. Acta.

[3] Halliwell B., Reznick Z.A., Packer L., Sen C.K., Holloszy J.O., Jackson M.J. (1998). Free radicals and oxidative damage in biology and medicine: An introduction. Oxidative Stress in Skeletal Muscle.

[4] Kettle A.J., Winterbourn C.C. (1997). Myeloperoxidase: A key regulator of neutrophil oxidant production. Redox Rep..

[5] Tobler A., Koeffler H.P., Harris J.R. (1991). Myeloperoxidase: Localization, structure, and function. Blood Cell Biochemistry.

[6] Fagerhol M.K., Dale I., Andersson T. (1980). Release and quantitation of a leukocyte derived protein (L1). Scand. J. Haematol..

[7] Johne B., Fagerhol M.K., Lyberg T., Prydz H., Brandtzæg P., Naess-Andresen C.F., Dale I. (1997). Functional and clinical aspects of the myelomonocyte protein calprotectin. J. Clin. Pathol.-Mol. Pathol..

[8] Nacken W., Roth J., Sorg C., Kerkhoff C. (2003). S100A9/S100A8: Myeloid representatives of the S100 protein family as prominent players in innate immunity. Microsc. Res. Tech..

[9] Donato R. (2001). S100: A multigenic family of calcium-modulated proteins of the EF-hand type with intracellular and extracellular functional roles. Int. J. Biochem. Cell Biol..

[10] Corbin B.D., Seeley E.H., Raab A., Feldmann J., Miller M.R., Torres V.J., Anderson K.L., Dattilo B.M., Dunman P.M., Gerads R., Caprioli R.M., Nacken W., Chazin W.J., Skaar E.P. (2008). Metal chelation and inhibition of bacterial growth in tissue abscesses. Science.

[11] Foell D., Wittkowski H., Vogl T., Roth J. (2007). S100 proteins expressed in phagocytes: A novel group of damage-associated molecular pattern molecules. J. Leukoc. Biol..

[12] Bøyum A., Løvhaug D., Seeberg E., Nordlie E.M. (1994). Identification of cytidine deaminase as inhibitor of granulocyte-macrophage colony formation. Exp. Hematol..

[13] Gran C., Bøyum A., Johansen R.F., Løvhaug D., Seeberg E.C. (1998). Growth inhibition of granulocyte-macrophage colony-forming cells by human cytidine deaminase requires the catalytic function of the protein. Blood.

[14] Bøyum A., Rønsen O., Tennfjord V.A., Tollefsen S., Haugen A.H., Opstad P.K., Bahr R. (2002). Chemiluminescence response of granulocytes from elite athletes during recovery from one or two intense bouts of exercise. Eur. J. Appl. Physiol..

[15] Bøyum A., Tennfjord V.A., Gran C., Løvhaug D., Øktedalen O., Brandtzæg P. (2000). Bioactive cytidine deaminase,an inhibitor of granulocyte-macrophage colony-forming cells,is massively released in fulminant meningococcal sepsis. J. Infect. Dis..

[16] Briheim G., Stendahl O., Dahlgren C. (1984). Intracellular and extracellular events in luminol-dependent chemi-luminescence of polymorphonuclear leukocytes. Infect. Immun..

[17] Yildiz G., Demiryürek A.T., Sahin-Erdemli I., Kanzik I. (1998). Comparison of antioxidant activities of aminoguanidine, methylguanidine and guanidine by luminol-enhanced chemiluminescence. Br. J. Pharmacol..

[18] Mates J.M. (2000). Effects of antioxidant enzymes in the molecular control of reactive oxygen species toxicology. Toxicology.

[19] Harrison C.A., Raftery M.J., Walsh J., Alewood P., Iismaa S.E., Thliveris S., Geczy C.L. (1999). Oxidation regulates the inflammatory properties of the murine S100 protein S100A8. J. Biol. Chem..

[20] Test S.T., Weiss S.J. (1984). Quantitative and temporal characterization of the extracellular H2O2 pool generated by human neutrophils. J. Biol. Chem..

[21] Valko M., Leibfritz D., Moncol J., Cronin M.T., Mazur M., Telser J. (2007). Free radicals and antioxidants in normal physiological functions and human disease. Int. J. Biochem. Cell Biol..

[22] Freinbichler W., Bianchi L., Colivicchi M.A., Ballini C., Tipton K.F., Linert W., Corte L.D. (2008). The detection of hydroxyl radicals *in vivo*. J. Inorg. Biochem..

[23] Ste-Marie L., Boismenu D., Vachon L., Montgomery J. (1996). Evaluation of sodium 4-hydroxybenzoate as an hydroxyl radical trap using gas chromatography-mass spectrometry and high-performance liquid chromatography with electrochemical detection. Anal. Biochem..

[24] Myhre O., Vestad T.A., Sagstuen E., Aarnes H., Fonnum F. (2000). The effects of aliphatic (n-nonane), naphtenic (1,2, 4-trimethylcyclohexane), and aromatic (1,2,4-trimethylbenzene) hydrocarbons on respiratory burst in human neutrophil granulocytes. Toxicol. Appl. Pharmacol..

[25] Steinbeck M.J., Khan A.U., Karnovsky M.J. (1993). Extracellular production of singlet oxygen by stimulated macrophages quantified using 9,10-diphenylanthracene and perylene in a polystyrene film. J. Biol. Chem..

[26] Halliwell B., Gutteridge J.M. (1984). Oxygen toxicity, oxygen radicals, transition metals and disease. Biochem. J..

[27] Dahlgren C., Briheim G. (1985). Comparison between the luminol-dependent chemiluminescence of polymorphonuclear leukocytes and of the myeloperoxidase-hooh system - influence of pH, cations and protein. Photochem. Photobiol..

[28] Albrecht D., Jungi T.W. (1993). Luminol-enhanced chemiluminescence induced in peripheral blood-derived human phagocytes: Obligatory requirement of myeloperoxidase exocytosis by monocytes. J. Leukoc. Biol..

[29] Gyllenhammar H. (1989). Effects of extracellular pH on neutrophil superoxide anion production, and chemiluminescence augmented with luminol, lucigenin or DMNH. J. Clin. Lab Immunol..

[30] Segal A.W., Geisow M., Garcia R., Harper A., Miller R. (1981). The respiratory burst of phagocytic cells is associated with a rise in vacuolar pH. Nature.

[31] Yousefi R., Imani M., Ardestani S.K., Saboury A.A., Gheibi N., Ranjbar B. (2007). Human calprotectin: Effect of calcium and zinc on its secondary and tertiary structures, and role of pH in its thermal stability. Acta Biochim. Biophys. Sinica.

[32] Trush M.A., Wilson M.E., van Dyke K., de Luca M.A. (1978). The generation of chemiluminescence (CL) by phagocytic cells. Methods in Enzymology.

[33] Brestel E.P. (1985). Co-oxidation of luminol by hypochlorite and hydrogen peroxide implications for neutrophil chemi-luminescence. Biochem. Biophys. Res. Commun..

[34] Arnhold J., Mueller S., Arnold K., Grimm E. (1991). Chemiluminescence intensities and spectra of luminol oxidation by sodium hypochlorite in the presence of hydrogen peroxide. J. Biolumin. Chemilumin..

[35] Stendahl O., Coble B.I., Dahlgren C., Hed J., Molin L. (1984). Myeloperoxidase modulates the phagocytic-activity of polymorphonuclear neutrophil leukocytes. Studies with cells from a myeloperoxidase-deficient patient. J. Clin. Invest..

[36] Sroussi H.Y., Berline J., Palefsky J.M. (2007). Oxidation of methionine 63 and 83 regulates the effect of S100A9 on the migration of neutrophils *in vitro*. J. Leukoc. Biol..

[37] Joshi S., Husain M.M., Chandra R., Hasan S.K., Srivastava R.C. (2005). Hydroxyl radical formation resulting from the interaction of nickel complexes of L-histidine, glutathione or L-cysteine and hydrogen peroxide. Hum. Exp. Toxicol..

[38] Bonomini F., Tengattini S., Fabiano A., Bianchi R., Rezzani R. (2008). Atherosclerosis and oxidative stress. Histol. Histopathol..

[39] Ehrchen J.M., Sunderkotter C., Foell D., Vogl T., Roth J. (2009). The endogenous Toll-like receptor 4 agonist S100A8/S100A9 (calprotectin) as innate amplifier of infection, autoimmunity, and cancer. J. Leukoc. Biol..

[40] Lim S.Y., Raftery M.J., Goyette J., Hsu K., Geczy C.L. (2009). Oxidative modifications of S100 proteins: Functional regulation by redox. J. Leukoc. Biol..

[41] Lim S.Y., Raftery M., Cai H., Hsu K., Yan W.X., Hseih H.L., Watts R.N., Richardson D., Thomas S., Perry M., Geczy C.L. (2008). S-nitrosylated S100A8: Novel anti-inflammatory properties. J. Immunol..

[42] Korndörfer I.P., Brueckner F., Skerra A. (2007). The crystal structure of the human (S100A8/S100A9)2 heterotetramer, calprotectin, illustrates how conformational changes of interacting alpha-helices can determine specific association of two EF-hand proteins. J. Mol. Biol..

[43] Hystad M.E., Myklebust J.H., Bø T.H., Sivertsen E.A., Rian E., Forfang L., Munthe E., Rosenwald A., Chiorazzi M., Jonassen I., Staudt L.M., Smeland E.B. (2007). Characterization of early stages of human B cell development by gene expression profiling. J. Immunol..

[44] Cadenas E., Davies K.J.A. (2000). Mitochondrial free radical generation, oxidative stress, and aging. Free Rad. Biol. Med..

[45] McCormick M.M., Rahimi F., Bobryshev Y.V., Gaus K., Zreiqat H., Cai H., Lord R.S., Geczy C.L. (2005). S100A8 and S100A9 in human arterial wall. Implications for atherogenesis. J. Biol. Chem..

[46] Dale I., Fagerhol M.K., Naesgaard I. (1983). Purification and partial characterization of a highly immunogenic human leukocyte protein, the L1-antigen. Eur. J. Biochem..

[47] Teigelkamp S., Bhardwaj R.S., Roth J., Meinardus-Hager G., Karas M., Sorg C. (1991). Calcium-dependent complex assembly of the myeloic differentiation proteins Mrp-8 and Mrp-14. J. Biol. Chem..

[48] Ryckman C., Vandal K., Rouleau P., Talbot M., Tessier P.A. (2003). Proinflammatory activities of S100: Proteins S10OA8, S10OA9, and S100A8/A9 induce neutrophil chemotaxis and adhesion. J. Immunol..

[49] Bøyum A., Løvhaug D., Tresland L., Nordlie E.M. (1991). Separation of leukocytes-improved cell purity by fine adjustments of gradient medium density and osmolality. Scand. J. Immunol..

